# Metabolic engineering of *Halomonas elongata*: Ectoine secretion is increased by demand and supply driven approaches

**DOI:** 10.3389/fmicb.2022.968983

**Published:** 2022-08-25

**Authors:** Karina Hobmeier, Martin Oppermann, Natalie Stasinski, Andreas Kremling, Katharina Pflüger-Grau, Hans Jörg Kunte, Alberto Marin-Sanguino

**Affiliations:** ^1^Professorship for Systems Biotechnology, Technical University of Munich, Garching, Germany; ^2^Division Biodeterioration and Reference Organisms, Bundesanstalt für Materialforschung und-prüfung (BAM), Berlin, Germany; ^3^Departament de Ciències Mèdiques Bàsiques, Universitat de Lleida, Lleida, Spain

**Keywords:** biochemistry, biotechnology, ectoine, *Halomonas elongata*, halophiles, metabolic engineering, microbiology

## Abstract

The application of naturally-derived biomolecules in everyday products, replacing conventional synthetic manufacturing, is an ever-increasing market. An example of this is the compatible solute ectoine, which is contained in a plethora of treatment formulations for medicinal products and cosmetics. As of today, ectoine is produced in a scale of tons each year by the natural producer *Halomonas elongata*. In this work, we explore two complementary approaches to obtain genetically improved producer strains for ectoine production. We explore the effect of increased precursor supply (oxaloacetate) on ectoine production, as well as an implementation of increased ectoine demand through the overexpression of a transporter. Both approaches were implemented on an already genetically modified ectoine-excreting strain *H. elongata* KB2.13 (Δ*teaABC* Δ*doeA*) and both led to new strains with higher ectoine excretion. The supply driven approach led to a 45% increase in ectoine titers in two different strains. This increase was attributed to the removal of phosphoenolpyruvate carboxykinase (PEPCK), which allowed the conversion of 17.9% of the glucose substrate to ectoine. For the demand driven approach, we investigated the potential of the TeaBC transmembrane proteins from the ectoine-specific Tripartite ATP-Independent Periplasmic (TRAP) transporter as export channels to improve ectoine excretion. In the absence of the substrate-binding protein TeaA, an overexpression of both subunits TeaBC facilitated a three-fold increased excretion rate of ectoine. Individually, the large subunit TeaC showed an approximately five times higher extracellular ectoine concentration per dry weight compared to TeaBC shortly after its expression was induced. However, the detrimental effect on growth and ectoine titer at the end of the process hints toward a negative impact of TeaC overexpression on membrane integrity and possibly leads to cell lysis. By using either strategy, the ectoine synthesis and excretion in *H. elongata* could be boosted drastically. The inherent complementary nature of these approaches point at a coordinated implementation of both as a promising strategy for future projects in Metabolic Engineering. Moreover, a wide variation of intracelllular ectoine levels was observed between the strains, which points at a major disruption of mechanisms responsible for ectoine regulation in strain KB2.13.

## 1. Introduction

The osmoadaptation strategy used by many bacteria and methanogenic archaea is the intracellular accumulation of osmolytes to balance the turgor pressure experienced in saline environments (Galinski, 1995). These osmoprotective organic compounds are called “compatible solutes” due to their compatibility with cell metabolism even at high concentrations (Brown, 1976). One of the most widespread compatible solutes is the aspartate-derivative ectoine (1,4,5,6,tetra-2-methyl-4-pyrimidonecarboxylic acid) (Galinski et al., 1985). Ectoine is biotechnologically relevant as protectant and stabilizer of proteins and other biomolecules against a wide range of adverse environmental factors like salinity but also heat, desiccation, freezing, thawing, and ionizing radiation. In addition to its function as a bioprotector, ectoine and its derivative hydroxyectoine have been proposed as potential drugs for diseases, such as Alzheimer's and rhinoconjunctivitis (Kanapathipillai et al., 2005; Salapatek et al., 2011; Bilstein et al., 2021). Moreover, its stabilizing effect was also observed on whole cells against stresses like radiation or cytotoxins (Lippert and Galinski, 1992; Kempf and Bremer, 1998; Pastor et al., 2010; Schröter et al., 2017). For these reasons, around 15,000 tons of ectoine are produced every year, and its price is close to 1,000 USD/Kg (Liu et al., 2021). The moderate halophilic gammaproteobacterium *Halomonas elongata* DSM 2581 (Vreeland et al., 1980) is able to grow at elevated salt concentrations due to the *de novo* synthesis of ectoine as its main compatible solute. A variety of strains of *H. elongata* are used as cell factories to produce ectoine for pharmaceutical and cosmetical use (Lentzen and Schwarz, 2006; Kunte et al., 2014) and a variety of other products (Ye and Chen, 2021). Additionally, there is an active interest in finding or creating other bacteria able to produce ectoine (Gießelmann et al., 2019; Wang et al., 2021).

Bioprocesses must often compete with chemical synthesis based on cheap petrochemicals, this creates a constant pressure to keep optimizing the overall process and streamlining metabolism to maximize the achievable product yield. Manipulating metabolic fluxes is not trivial due to the abundant and often unknown mechanisms by which the cell regulates them to meet its needs. The complexity of metabolic regulation and the need to address it at a systems level was recognized long time ago (Savageau, 1971, 1976) and confirmed by the failure of early attempts to increase metabolic fluxes by directly overexpressing a few key enzymes (Ruijter et al., 1997). Even nowadays, regulatory feedback loops are common obstacles metabolic engineering has to deal with Yu et al. (2021). Early concepts dealing with metabolism as a system based on supply and demand blocks (Hofmeyr and Cornish-Bowden, 2000) highlighted the need to look beyond the pathways, into the global economy of the cell. In the case of ectoine, its intracellular concentration is tightly controlled to balance the external salt concentration (Dötsch et al., 2008; Czech et al., 2018). Since artificial increases in metabolic fluxes producing ectoine are very likely to be countered by feedback mechanisms, rational manipulation of the fluxes is a challenging but feasible approach to improve ectoine production (Ma et al., 2020). Unlike the well studied end-product inhibition pattern commonly found in amino acid synthesis (Savageau, 1975; Alves and Savageau, 2000), whatever mechanism the cell uses for ectoine homeostasis needs to ensure ectoine concentration to be adjustable across a broad range to enable adaptation to different salinities and other environmental conditions. Although the detailed mechanisms controlling ectoine synthesis have not been completely elucidated, it is known that *H. elongata* prioritizes the uptake of compatible solutes from the medium over *de novo* ectoine synthesis and it has been proposed that the export of ectoine to the periplasmic space and subsequent re-uptake into the cytoplasm may be part of the regulatory loop (Grammann et al., 2002; Kunte, 2006). The existence of this traffic of ectoine between compartments and its nature as an aspartate derivative places ectoine synthesis within a tightly regulated metabolic environment.

Since the whole aspartate family of amino acids has oxaloacetate as a precursor, the *de novo* synthesis of ectoine elevates the demand for it and withdraws carbon from the TCA cycle. The anaplerotic node that is responsible for the replenishment of TCA cycle intermediates gains a special position in the *H. elongata* central metabolism when grown on glycolytic carbon sources. The anaplerotic role is shared between the phosphoenolpyruvate carboxylase (Ppc) and the reversible membrane-associated Na^+^-pump oxaloacetate decarboxylase (Oad). Ppc is thermodynamically more favorable, but Oad is directly coupled to the sodium driving force, ensuring a sufficient increase in anaplerotic flux for ectoine synthesis depending on the sodium gradient with this alternative pathway (Hobmeier et al., 2020). The remaining enzymes involved in this part of the metabolic network, to which we will refer from now on as the PEP-PYR-OAA node, are the PEP carboxykinase (PckA) and the malic enzymes (MaeA and MaeB). PckA catalyzes the irreversible decarboxylation of oxaloacetate to phosphoenol-pyruvate, consuming ATP in the process (Sauer and Eikmanns, 2005). The PckA-mediated reaction is really only necessary when the cell grows on gluconeogenetic carbon sources, but it has been shown to be active in other bacteria during glycolytic growth as well (Chao and Liao, 1994; Yang et al., 2003). The futile cycle created by the simultaneous activity of Ppc and PckA is a sink for ATP and prevents accumulation of oxaloacetate, making PckA a promising target for flux optimization. The malic enzymes are normally also gluconeogenetic enzymes converting malate to pyruvate (Sauer and Eikmanns, 2005). In *H. elongata*, there are two isoenzymes present, namely *maeA* (HELO_3817), which corresponds to *sfcA* in *E. coli*, and *maeB* (HELO_3763). They have different cofactor specificities with *maeA* being linked to NAD and *maeB* to NADP. For both isoenzymes an oxaloacetate-decarboxylating activity is described as possible in the literature, meaning not only malate but also oxaloacetate can be used as a substrate (Sauer and Eikmanns, 2005). This again constitutes a competing pathway for ectoine synthesis. However, enzyme assays only showed *in vitro* activity for the NADP-dependent isoenzyme MaeB.

Besides accumulating in the cytoplasm and being diluted by growth, ectoine has other possible metabolic fates. Ectoine can be used as a carbon source by *H. elongata* through an independent degradation pathway (Schwibbert et al., 2011), or it can also be exported to the periplasm through a not yet identified transporter (Vandrich et al., 2020) and then taken up through the ectoine-specific TRAP transporter encoded in the *teaABC* operon. The discovery of this cycle was possible through the modified “*leaky mutant”* strain KB2.13 (*H. elongata* DSM 2581, Δ*teaABC* Δ*doeA*) which, true to its name, leaks ectoine into its surroundings (Kunte et al., 2002; Kunte, 2006) due to the deletion of the *teaABC* operon (Grammann et al., 2002; Kunte et al., 2014). The additional deletion of the ectoine hydrolase gene *doeA* abolishes ectoine degradation and prevents this strain from consuming ectoine (Schwibbert et al., 2011). It is noteworthy that the leaky phenotype can already be observed after solely removing the periplasmic substrate-binding protein (SBP), TeaA, but leaving the small and large transmembrane subunits (TeaB and TeaC) intact. Since the TRAP is a secondary transporter and it is not driven by ATP hydrolysis, but a by an ion gradient (Kunte et al., 2002), both transmembrane proteins could potentially facilitate the bidirectional transport of ectoine in the absence of the SBP TeaA.

The aim of this work was to explore the potential for increased ectoine production in the industrial producer strain *H. elongata*. To achieve this goal, we used two complementary strategies that aim at a rational manipulation of the relevant fluxes: increasing the supply of precursors and boosting the demand for the end-product.

## 2. Materials and methods

### 2.1. *H. elongata* strains and growth experiments

The *H. elongata* strains used in this work are derived from the modified strain *H. elongata* KB2.13 (Δ*teaABC* Δ*doeA*; Kunte et al., 2002; Kunte, 2006), and are listed in Table 1. Further modifications were introduced using homologous recombination as detailed below. All *H. elongata* strains were routinely grown at 30°C and under shaking at 220 r.p.m. in liquid media LB (Miller) enriched with 1 M NaCl or MM63 minimal medium [KH_2_PO_4_ 100 mM, (NH_4_)_2_SO_4_ 15 mM, KOH 75 mM, NaCl variable (from 0.17 to 2 M depending on the experiment), carbon source (glucose or acetate depending on the experiment) 27.75 mM, MgSO_4_ ·7 H_2_O 1 mM, FeSO_4_ ·7 H_2_O 0.004 mM] (Larsen et al., 1987). In general, three biological replicates were always used in each growth experiment for each strain and condition except for the ectoine secretion experiments with TeaBC, TeaB, and TeaC. Here, six replicates were used in the pre-culture steps in order to induce the heterologous gene expression in three while leaving three others uninduced as references.

**Table 1:** Summary of all modified strains characterized in this work based on the parental strain KB2.13 (*H. elongata* DSM 2581, Δ*teaABC* Δ*doeA*). Most experiments presented in this work were performed with the in frame null mutations, only the screening in microtitter plate was done with the marker replacement strains for convenience. No phenotypic differences were observed between the marker replacement strains and their corresponding in-frame full deletion strains.

**Strain**	**Genotype *H. elongata* KB2.13**	**Type of modification**	**Phenotype compared to KB2.13**
KH1.1	Δ*pckA*	In-frame null mutation	Reduced growth rate on glucose, no differences growing on acetate, increased ectoine excretion (compared to KB2.13)
KH1.2	Δ*pckA*::Sm^*R*^	Marker replacement of PckA	Resistant against streptomycin, reduced growth rate on glucose especially at low salinities but shorter lag phase than KH1.1, no differences growing on acetate
KH2.1	Δ*pckAΔmaeB*	In-frame null mutation	Reduced growth rate on glucose especially at low salinities, no differences growing on acetate, same ectoine synthesis as KH1.1
KH2.2	Δ*pckA* Δ*maeB*::Sm^*R*^	Marker replacement of *maeB*	Resistant against streptomycin, reduced growth rate on glucose especially at low salinities, no differences growing on acetate
KH3.1	Δ*pckA*Δ*ppc*	In-frame null mutation	Reduced growth rate on glucose especially at low salinities, no differences growing on acetate, higher ectoine excretion than KB2.13

In the first step of the growth experiments, for each biological replicate a single colony was taken from a solid agar plate and grown in 3 mL liquid LB medium enriched with 1 M NaCl. Subsequently, an aliquot of this overnight culture was taken to inoculate 3 mL MM63 minimal medium with 1 M NaCl and either glucose or acetate as carbon source in a 1:100 ratio. This culture was then again used to inoculate a subsequent MM63 culture with an adjusted inoculum volume to achieve an OD_600_ of 0.01 in the new culture. This third pre-culture differs slightly from experiment to experiment since it is used to adjust the cultures to the respective main culture medium. In case of the microtiter plate screenings, four 3 mL MM63 minimal medium cultures with each containing a different NaCl concentration (0.17, 0.5, 1, and 2 M) and the carbon source used in the previous step were inoculated. For all shake flask experiments with the general MM63 minimal medium (not SO_4_-limited medium) the same medium as in the previous pre-culture step (1 M NaCl) was used. for the ectoine excretion experiment with SO_4_-limited medium, a SO_4_-limited MM63 minimal medium with 1 M NaCl and glucose was used to inoculate another 3 mL pre-culture step in MM63 SO_4_-limited minimal medium with glucose, until the final inoculation of the main culture. During the transfers, the cultures were kept in exponential growth at all times and the final transfer to the main culture was performed with an adjusted inoculation volume reaching an initial OD_600_ of 0.01 in the main culture medium.

The screening experiments were carried out in sterile 96-well plates (Greiner, Germany) with a filling volume of 0.2 mL per well and the four NaCl concentrations (0.17, 0.5, 1, and 2 M) already used in the pre-culture for each replicate. As a blank as well as sterile control, wells with the sterile medium were measured in parallel. The measurements were performed in an automated microplate reader (Tecan, Austria) at 30°C, which was set to shake briefly and measure the OD_600_ in regular intervals every 10 min. The OD_600_ evolution was followed for a time frame of ~16–24 h until the stationary phase was reached.

Shake flask experiments were routinely performed in 500 mL flasks with 10% working volume incubated in a rotary shaker. The OD_600_ was followed using a spectrometer (Eppendorf, Germany). Ectoine samples were either taken in the late exponential phase as external concentration in relation to the biomass (g/gDW) or after complete consumption of the carbon source as titer (g/L). In the ectoine secretion experiments specifically, after inoculation the cultures were grown to an OD_600_ of 0.1 as an adaptation phase, which lasted ~7 h. After reaching OD_600_ 0.01 half of the cultures were induced with 0.1 mM 3-methylbenzoic acid (3-MB) diluted in ethanol (EtOH). The same volume of EtOH solvent, which was used for induction, was added to the remaining uninduced references without 3-MB. During the overexpression of TeaBC, 1 mL samples for ectoine detection were taken at three time points in the late exponential phase. In the experiment overexpressing TeaB and TeaC individually, the ectoine was measured at one time point in the late exponential phase as well as the titers.

### 2.2. Genome modification using homologous recombination

The genome modifications, marker replacement with a Sm^*R*^ cassette (*aadA*) from pSEVA434 (Silva-Rocha et al., 2013) and in-frame null mutations, were performed using homologous recombination (Martínez-García and de Lorenzo, 2011). The method established for *Pseudomonas putida* was adapted as detailed in Hobmeier et al. (2020). First an integration vector (Supplementary Table 1) specific for the modification and targeted gene was constructed *via* Gibson Assembly using the oligonucleotides specified in Supplementary Table 1. For the deletion of *ppc*, the previously constructed plasmid pSEVA_Δppc described in Hobmeier et al. (2020) was used. After generating the integration vector, it was transferred into the *H. elongata* respective strain by triparental mating. After successful integration of the vector into the genome a second conjugal transfer of the expression plasmid pSW-2 for the homing endonuclease I-SceI was carried out. Subsequently, the recombination event was triggered by induction of I-SceI expression, which causes double-strand breaks in the genome at the specific recognition sites introduced together with the integration plasmid. The mutant strains were selected based on the desired phenotype and the correct genotype was verified using polymerase chain reaction (PCR) and sequencing (Eurofins Genomics, Germany). All used enzymes were purchased from New England Biolabs (USA).

### 2.3. Construction of the expression vectors for TeaBC, TeaB, and TeaC

The expression vectors were constructed *via* Gibson Assembly using the oligonucleotides specified in Supplementary Table 1. The oligonucleotides were designed to regenerate the restriction sites used to linearize the plasmid pSEVA438 (Silva-Rocha et al., 2013) which was used as backbone. pSEVA438 already harbors the inducible XylS/Pm promoter with an empty multiple cloning site. The plasmid was linearized using the restriction sites *SacI* and *HindIII* for the expression plasmids pSEVA438-*teaBC* and pSEVA438-*teaC*. For pSEVA438-*teaB* the combination *SacI* and *PstI* was used. The inserts were generated by PCR amplification using the protocol specified by the manufacturer. Using the oligonucleotides, the synthetic ribosomal binding site (5' aggaggcttcat 3') was inserted for each construct to facilitate translation. The Gibson Assembly reaction was carried out as specified in the manufacturer's protocol and transformed into TSS-competent *E. coli* DH5α λ*pir* cells (Chung et al., 1989) described in detail by Hobmeier et al. (2020). The correct genotypes were verified using PCR and sequencing (Eurofins Genomics, Germany). All applied enzymes were purchased from New England Biolabs (USA).

### 2.4. Transcriptomic analysis

The collection of RNA for transcriptomic analysis was part of the experiment described in Hobmeier et al. (2022). In this work, we add the data for strain KH1.1, which has not been published before. The parental strain KB2.13 (*H. elongata* DSM 2581 Δ*teaABC* Δ*doeA*) and derivative KH1.1 (*H. elongata* KB2.13 Δ*pckA*) were grown in 50 mL scale as described earlier in MM63 minimal medium with 1 M NaCl and glucose. After reaching an OD_600_ of ~0.5, well within the exponential growth phase, samples were taken and treated with RNAprotect reagent (Qiagen, Germany). Subsequently, the RNA was isolated using the Macherey-Nagel NucleoSpin RNA kit (Macherey-Nagel, Germany) and sent to GATC Biotech company (Germany). They generated a strand-specific cDNA library and performed the RNA-sequencing at the company facilities using the Illumina NovaSeq 6000 S4 XP. The obtained FASTQ files were comprised of paired-end reads with lengths of 150 bp. The data was further processed as described in Hobmeier et al. (2022). The resulting transcript per million (TPM) were then analyzed regarding differential expression of genes based on a log2-fold change (log2-FC) of |1.5| between strains using Python scripts. For clustering, Principal Component Analysis (PCA) was applied to the set of TPM counts for all conditions: wild type *H. elongata* DSM 2581 (Wt) on glucose and acetate, KB2.13 strain on glucose and KH1.1 on glucose (three replicates each). In order to cluster genes by their response to different conditions and not by their overall level of expression, the first principal component was not used to compute distances in the clustering process.

### 2.5. Ectoine detection via RP-HPLC

The quantification of extracellular ectoine was performed using reverse-phase (RP) high performance liquid chromatography (HPLC) analysis, which has been described in detail in Hobmeier et al. (2020). However, since the secreted ectoine in the medium was measured no extraction was needed. After sampling, the biomass was separated by centrifugation for 5 min at 15,000 × g and 25°C and the supernatant was carefully transferred into a new tube. The supernatant was then diluted 1:10 with the mobile phase (acetonitrile/phosphate) and analyzed using a reverse phase column (Nucleodur 100 5 NH2 RP CC 125/4, Macherey-Nagel). With a UV-detector the absorption of ectoine at a wavelength of 210 nm was recorded. Ectoine samples were taken either in the late exponential phase or after the end of the batch process. Samples taken during the growth phase were normed using the biomass at the time of sampling (gram per gram dry weight, g/gDW) by applying the previously determined OD_600_ to ash free dry weight correlation published by Hobmeier et al. (2020). Because the measured OD_600_ values after reaching the stationary phase are not reliable the amount of produced ectoine at the end of the process was determined as the final ectoine titer (g/L).

## 3. Results

### 3.1. Growth and salt tolerance of modified strains

**Figure 1:**
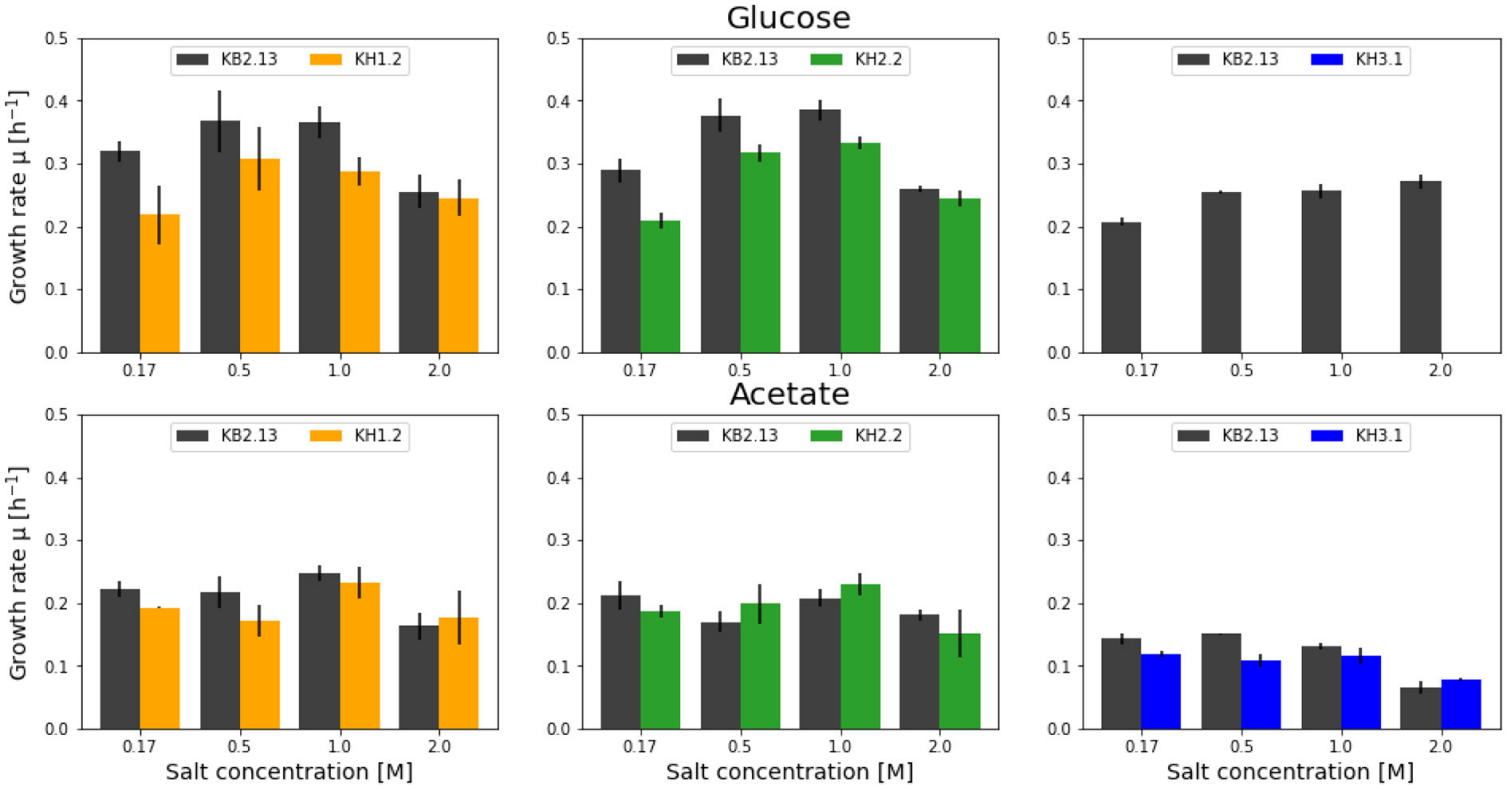
Overview of the growth rates achieved by the modified strains KH1.2 (KB2.13, Δ*pckA*::Sm^*R*^) (orange), KH2.2 (KB2.13, Δ*pckA* Δ*maeB*::Sm^*R*^) (green), and KH3.1 (KB2.13, Δ*pckAΔppc*) (blue) in microtiter plate screenings growing on glucose (top) and acetate (bottom). For each screening the parental strain, KB2.13 (*Halomonas elongata* DSM 2581, Δ*teaABC* Δ*doeA*) (gray) is grown in parallel as reference. The deleted genes *pckA, ppc* and *maeB* encode phosphoenolpyruvate carboxykinase, phosphoenolpyruvate carboxylase, and the NADP-dependent malic enzyme, respectively.

We screened various *H. elongata* knockout mutants with modifications in the PEP-PYR-OAA node in comparison to KB2.13, from which they are derived. In detail, these strains are KH1.2 with a marker replacement in phosphoenolpyruvate carboxykinase (Δ*pckA*::Sm^*R*^), KH2.2 with a null mutation in *pckA* and a marker replacement in NADP-dependent malic enzyme (Δ*pckA*Δ*maeB*::Sm^*R*^), and finally KH3.1 with null mutations in phosphoenolpyruvate carboxykinase and phosphoenolpyruvate carboxylase (Δ*pckA*Δ*ppc*). The impact on physiology and ectoine synthesis due to these modifications are explored in the following. The determined growth rates are shown in Figure 1. The reference strain (KB2.13) is illustrated in gray. The knockout mutants KH1.2, KH2.2, and KH3.1 are depicted in orange, green, and blue, respectively.

#### 3.1.1. KH1.2—Phosphoenolpyruvate carboxykinase deletion strain

In the literature, the PckA-mediated reaction is generally designated as a gluconeogenetic reaction. But the disruption in *H. elongata* KB2.13 had an impact on its growth behavior specifically with glucose as substrate. Across a range of salt concentrations, spanning from low salinity at 0.17 M NaCl, the plateau of salt optimum from ~0.5 to 1 M NaCl, and up to high salinity at 2 M NaCl, the KH1.2 strain grew significantly slower than the parental strain KB2.13 in all salt concentrations except high salt (2 M NaCl). In contrast, with the gluconeogenetic substrate acetate a significant reduction in growth rate was only observed at low salt (0.17 M NaCl).

#### 3.1.2. KH2.2—Phosphoenolpyruvate carboxykinase and NADP-dependent malic enzyme deletion strain

The main role of the NADP-dependent malic enzyme MaeB lies in the production of NADPH during growth on acetate (Wang et al., 2011). The growth rates of the double knockout strain KH2.2 was very similar to KB2.13. Growing on acetate no significant differences were found. The biggest impact of the *maeB* deletion occurred with glucose as carbon substrate. Only at low salinity (0.17 M NaCl) a reduced growth rate was observed.

#### 3.1.3. KH3.1—Phosphoenolpyruvate carboxykinase and phosphoenolpyruvate carboxylase deletion strain

Phosphoenolpyruvate carboxylase is one of the two enzymes carrying the anaplerotic flux in *H. elongata*. In KH3.1, both carboxylating and decarboxylating reactions between phosphoenolpyruvate and oxaloacetate are abolished. Therefore, glycolytic fluxes necessarily have to pass through the ATP-forming pyruvate kinase to pyruvate before anaplerosis is possible. For this strain, no growth with glucose could be determined up until 18 h after inoculation. However, in the previous pre-culture steps using the same growth medium growth on glucose was observed. It has already been shown in Hobmeier et al. (2020) that the removal of phosphoenolpyruvate carboxylase leads to a rather unstable phenotype with an increased lag phase and high variability in growth rates. KH3.1 was often unable to grow on glucose in microtiter plate. However, it grew well with acetate as carbon source, albeit with a reduced growth rate at lower salt concentrations of 0.17 and 0.5 M NaCl.

#### 3.1.4. Growth and ectoine homeostasis in batch cultures

The growth of the deletion mutants on glycolytic substrate was further verified in shake flask experiments at the salt optimum 1 M NaCl. The growth rates for the deletion strains KH1.1 and KH2.1 (in-frame null mutations) in relation to the parental strain KB2.13 was determined in four distinct batch experiments. KB2.13 grew significantly faster with an average growth rate of 0.467 ± 0.044 h^−1^. KH1.1 and KH2.1 both showed the same average growth rate of 0.345 ± 0.051 and 0.348 ± 0.057 h^−1^, respectively. Therefore, the reduced growth can be attributed directly to the loss of phosphoenolpyruvate carboxykinase. Also, for this mutant a longer lag phase was observed. Even though the additional deletion of *maeB* does not affect the growth rate, there is a noticeable impact on the lag phase. The prolonged lag phase observed for KH1.1 is shortened in KH2.1 and it can commence growth faster after inoculation.

The additional deletion of phosphoenolpyruvate carboxylase in strain KH3.1, was introduced to shed light on the phenotype of KH1.1 growing with glucose as carbon substrate. The growth deficit observed for KH3.1 with glucose was shown to be an artifact associated to cultivation on microtiter plate. However, compared to KB2.13 and KH1.1 the growth rate was found to be considerably reduced at only 0.238 ± 0.010 h^−1^ on glucose. Strain KH1.1 grew at a growth rate of 0.303 ± 0.036 h^−1^, and the fastest growth rate was as always observed for KB2.13 at 0.450 ± 0.004 h^−1^. The diminished growth rate after deletion of Ppc is not surprising since it is thought to carry a major portion of the anaplerotic flux during glycolytic growth even though the alternative Oad can take over a portion of the flux at the applied salt concentration 1 M NaCl (Hobmeier et al., 2020). In the late exponential phase, an intracellular ectoine content of 0.018 ± 0.002 g/gDW was determined for KB2.13 and a much higher content of 0.080 ± 0.016 g/gDW for KH1.1. The deletion of *ppc* led to a decrease in ectoine content to 0.051 ± 0.009 g/gDW. However, this is still a 2.8-fold increase compared to KB2.13. These important variations in ectoine concentration point toward a major disruption of the regulatory mechanisms that normally keep ectoine homeostasis. Growth on the gluconeogenetic substrate acetate was not expected to be affected by Ppc and indeed, all strains exhibited very similar growth rates on acetate, with growth rates of 0.237 ± 0.003, 0.227 ± 0.004, and 0.222 ± 0.004 h^−1^ for KB2.13, KH1.1, and KH3.1. This was also reflected in the intracellular ectoine content with 0.019 ± 0.002, 0.023 ± 0.001, and, again, 0.023 ± 0.001 g/gDW, respectively.

### 3.2. Ectoine excretion in KH1.1 (Δ*pckA*) and KH2.1 (Δ*pckA*Δ*maeB*)

**Figure 2:**
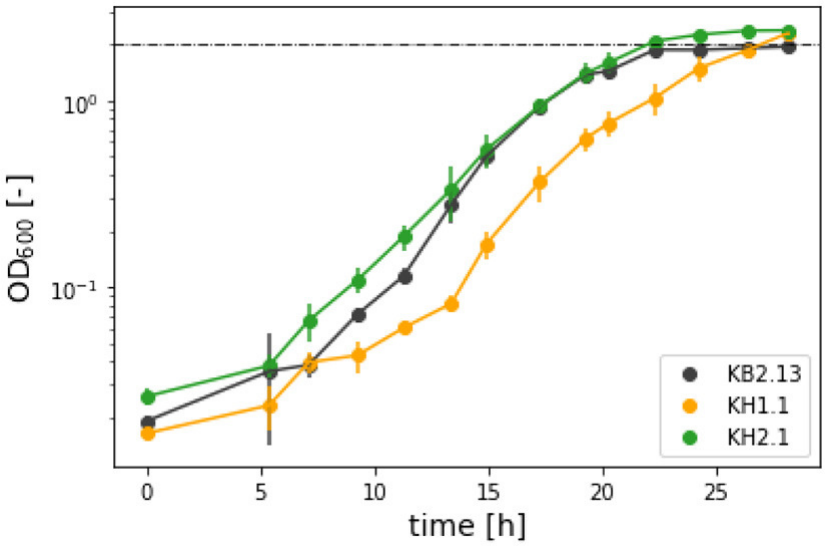
OD_600_ evolution in half-logarithmic depiction for the ectoine synthesis experiment in SO_4_-limited medium with the strains KB2.13 (*Halomonas elongata* DSM 2581, Δ*teaABC* Δ*doeA*) (gray), KH1.1 (KB2.13, Δ*pckA*) (orange), and KH2.1 (KB2.13, Δ*pckA* Δ*maeB*) (green).

**Figure 3:**
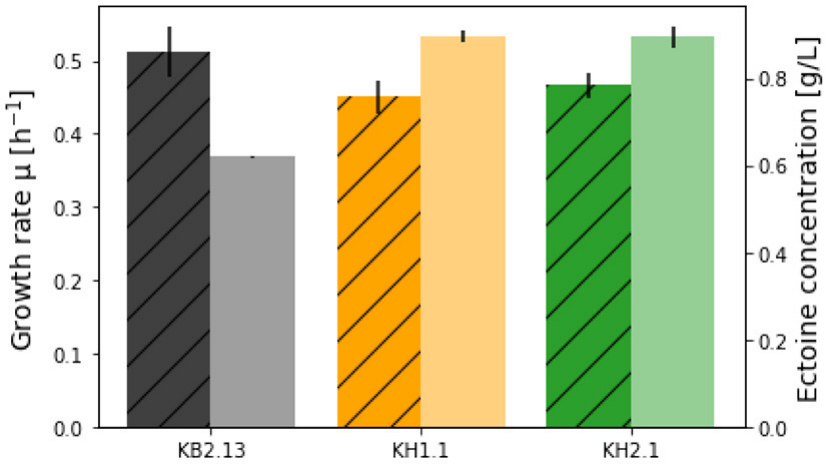
Growth rates (striped bars) and ectoine titers (no pattern) achieved in the SO_4_-limited medium with the strains KB2.13 (*Halomonas elongata* DSM 2581, Δ*teaABC* Δ*doeA*) (gray), KH1.1 (KB2.13, Δ*pckA*) (orange), and KH2.1 (KB2.13, Δ*pckA* Δ*maeB*) (green).

Ectoine analytics in shake flask experiments is challenging due to the low biomass achieved in such cultures, which leads to ectoine accumulation being at the lower end of the HPLC detection limit during the exponential growth phase. Since the genetic background of all the strains discussed in this work includes an impaired ectoine catabolic pathway (Δ*doeA*), it was possible to compare cultures grown into the stagnation phase. The titers after the end of the process are the maximal final concentrations achievable from the applied substrate. To further increase ectoine yields, an experiment in a SO_4_-limited medium was carried out. Based on a standard biomass formula of *CH*_1.6_*O*_0.37_*N*_0.26_*S*_0.006_ (Battley, 1991) the amount of sulfate in the medium was adjusted to limit the maximum biomass to an OD_600_ of 2. As can be seen in Figures 2, 3, the growth of KB2.13, KH1.1, and KH2.1 compared to each other coincided with the pattern observed in the regular minimal medium with KB2.13 growing significantly faster at 0.512 ± 0.035 h^−1^ and both modified strains at similar growth rates of 0.450 ± 0.024 h^−1^ for KH1.1 and 0.466 ± 0.017 h^−1^ for KH2.1. As mentioned before, the deletion of *maeB* in KH2.1 leads to a reduced lag phase, which is apparent in the growth curves depicted in Figure 3. Another interesting finding here is that the evolution of OD_600_ for KB2.13 stops precisely at the theoretically determined limit of OD_600_ 2, but the modified strains eventually exceeded the OD_600_ limit (Figure 2). This hints toward possible changes in the biomass composition. A tendency of the modified strains to accumulate PHB would be consistent with these results as well as previous observations of *H. elongata's* behavior under stress (Hobmeier et al., 2022). The available data on the transcriptome of KH1.1 do not provide enough evidence to confirm this since the upregulation of *phbC* in this strain amounts to a log2-fold-change of 1.3, which is close but still below the chosen threshold of 1.5 for differential expression.

After 48 h the final ectoine titers in the medium were measured for all strains. With 5 g/L glucose KB2.13 produced 0.619 ± 0.002 g/L. The modified strains reached considerably higher titers of 0.895 ± 0.012 g/L for KH1.1 and 0.894 ± 0.025 g/L for KH2.1. This equates to a 45 % increase in ectoine titer. The improvement is clearly caused by the disruption of the PckA futile cycle and the additional removal of NADP-ME has no impact on ectoine synthesis.

### 3.3. RNA-Seq analysis of modified strain KH1.1

**Figure 4:**
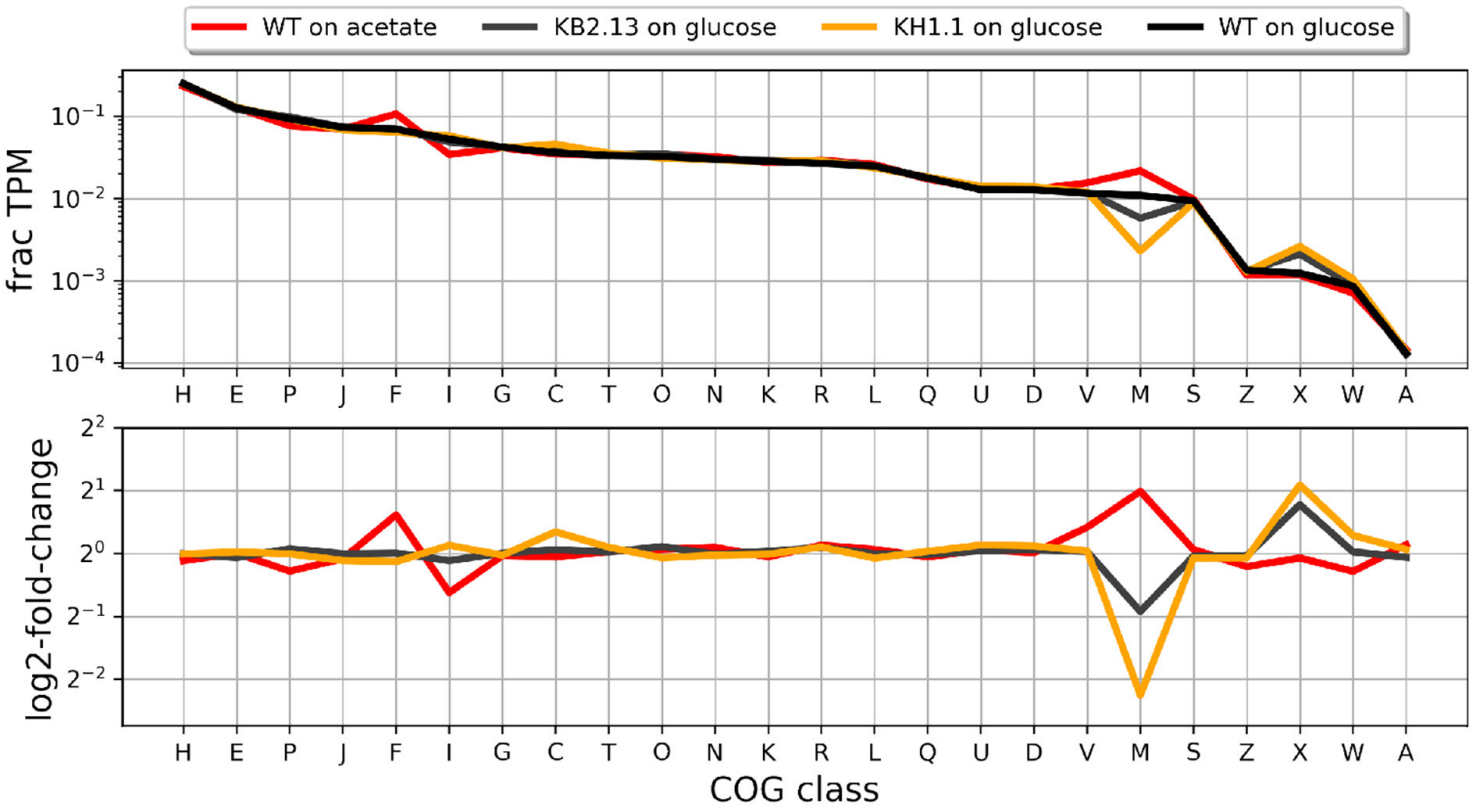
Fraction of the transcriptome occupied by each COG class in each experiment (upper panel) and log2-fold-change for each case with respect to the wild type *Halomonas elongata* DSM 2581 growing on glucose. The strains are KB2.13 (*Halomonas elongata* DSM 2581, Δ*teaABC* Δ*doeA*) (gray) and KH1.1 (KB2.13, Δ*pckA*) (orange).

During the RNA-Seq experiments described by Hobmeier et al. (2022), RNA was also collected from KH1.1. Figure 4 shows the distribution of COG classes in the transcriptome of this strain in relation to those of the previous publication. The profiles suggest that the changes in transcription levels grouped by COG class that appear in KB2.13 become more prominent after the additional deletion of *pckA* in strain KH1.1.

In general, the transcription profile of KH1.1 changes more with respect to the parental strain (KB2.13) than that strain did with respect to the wild type. Genes showing a log2-fold-change larger than 1.5 between strains, were clustered by their pattern of transcription as described in materials and methods (see Supplementary material for details). The two larger clusters were formed by genes involved in chemotaxis (33 genes) and flagellar motility (17 genes).

All these genes have been previously found to be already close or beyond the threshold of down-regulation in KB2.13 and also severely down-regulated by cells growing on low-salinity. The down-regulation of these genes in KH1.1 with respect to its parental strain KB2.13 is even more prominent. The next few clusters in size include a large number of uncharacterized or poorly annotated genes. For instance, the third largest cluster includes 17 genes which are poorly characterized except, for two genes involved in the degradation of ectoine: *doeB* and *doeC*. These genes are clearly down-regulated in KB2.13 (in which *doeA* is deleted) but seem unaffected by the deletion of *pckA* in the KH1.1 strain. The down-regulation in KB2.13 is most likely a direct consequence of the *doeA* deletion. The next cluster with 14 genes includes *teaD* (Schweikhard et al., 2010) which shows increased transcription in the KB2.13 strain and a series of genes that are up-regulated exclusively in KH1.1. The up-regulation of *teaD* can also be attributed to the introduced modifications in KB2.13. Due to the deletion of *teaABC* the adjacent open reading frame *teaD* directly underlies the *tea* operon promoters resulting in an artificial overexpression. The remaining genes only affected in KH1.1 include *acnA*, which encodes for the TCA cycle enzyme aconitase, the chaperone (*clpB*), the cold-shock protein (*cspA4*), and a APC family transporter (HELO_1536). Smaller clusters normally involve well annotated genes. Cluster 13 is formed by consecutive genes *putP* and *putA* involved in proline metabolism, as well as HELO_1459 coding for an OmpW family protein and HELO_2165A coding for a UspA domain protein. These genes are up-regulated in KH1.1 and also show a similar behavior in the wild type growing on acetate or in low salinity. Cluster 14 contains only two consecutive genes HELO_4326 and HELO_4327 coding for a tryptophan synthase and they are down-regulated both in strain KH1.1 and again in the wild type growing on acetate or in low salinity. A similar behavior is exhibited by the two genes in cluster 12: NAD-dependent acetaldehyde dehydrogenase HELO_2817 (*acoD*) and alcohol dehydrogenase HELO_2818 (*adh2*) except these two genes are up-regulated at low salinity. The genes for the multidrug efflux pump AcrAB (HELO_3739 and HELO_3738) is up-regulated exclusively in KH1.1. This transporter facilitates the energy-dependent vertical transport of diverse compounds from the cytoplasm directly into the extracellular space (Du et al., 2014).

### 3.4. Ectoine excretion *via* transmembrane proteins TeaB and/or TeaC

As a way to avoid potential feedback inhibition by ectoine on its own synthesis, we explored ways to enhance ectoine removal from the cytoplasm. This action on demand was implemented independently from the supply oriented method shown above, but both approaches are clearly complementary. The mechanisms that secretes ectoine to the periplasm has not yet been fully characterized. It has been established that 20% of this flux is carried by the mechanosensitive channels, but the remaining 80% goes through an as yet unidentified transporter (Vandrich et al., 2020). Therefore, the removal of ectoine was implemented using the channel building proteins of the ectoine specific TRAP transporter: TeaB and TeaC. Since this channel is a symporter of ectoine with sodium, ectoine export through this method involves overcoming the sodium gradient and adding a sodium export flux. The single knockout of the periplasmic substrate binding protein (SBP) TeaA already results in the same leaky phenotype as the complete removal of the *tea* operon (HELO_4274-6). Without the specific SBP the re-uptake of ectoine is disrupted and accumulates in the periplasm over time, leading to a constant loss of ectoine into the extracellular space.

We tested the induced overexpression of both membrane proteins in strain KB2.13, in which the *tea* operon is deleted and, thus, TeaA is not present. Three different plasmids based on the pSEVA architecture were assembled with *teaBC, teaB*, or *teaC* under the inducible XylS/Pm promoter. To rule out any impact of the inducer or solvent, their impact was also examined in KB2.13 but no significant differences in ectoine secretion between untreated cultures, cultures treated with only solvent (EtOH), and cultures treated with the inducer (0.1 mM 3-methyl-benzoic acid in EtOH) could be detected (see Supplementary material).

**Figure 5:**
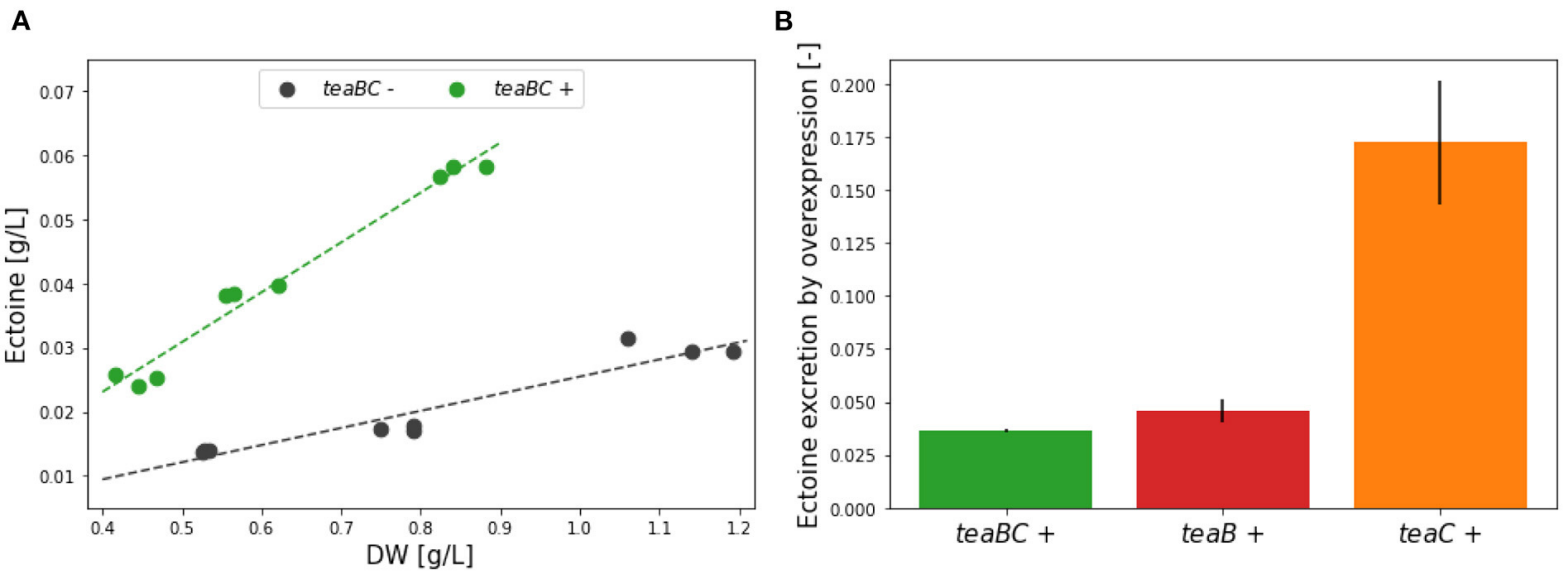
**(A)** Ectoine accumulation rates for the overexpression of *teaBC* (green, induced) and the uninduced control (gray) in the late exponential phase. The rates were calculated for three biological replicates at three time points. **(B)** Comparison of the accumulated ectoine in the medium in the late exponential phase after 13 h of process time for the overexpression of the genes encoding the transmembrane proteins: *teaBC* (green), *teaB* (red), and *teaC* (orange). All genes were introduced using pSEVA438 plasmids under the inducible XylS/Pm promoter (see text for details).

First, the effect of the complete transmembrane complex TeaBC was investigated. After inoculation, initially the cultures were left uninduced for a period of ~7 h in order for the cultures to adapt. Up to an OD_600_ of 0.1 all KB2.13 (pSEVA438-*teaBC*) replicates grew with the same growth rate of 0.468 ± 0.010 h^−1^. After induction of half of the cultures with 0.01 mM inducer at OD_600_ 0.01 this exponential growth continued for 3 h until the late exponential phase was reached after ~10 h. From then on, the uninduced cultures showed a growth rate of 0.299 ± 0.011 h^−1^ and the induced cultures a slightly slower (16.7%) growth rate of 0.249 ± 0.006 h^−1^. Regarding the ectoine concentration in the medium, the induced *teaBC* expressing cultures (green) during the late exponential phase accumulated ectoine at an increased rate of 0.078 g/L extracellular ectoine per g/L dry weight, in contrast to the uninduced cultures (black) with a rate of only 0.027 g/L (Figure 5A). This translates into an almost 3-fold increase of ectoine secretion caused by the TeaBC channels.

Cultures of KB2.13 harboring either the pSEVA438-*teaB* or pSEVA438-*teaC* expression plasmid were grown in parallel. After an initial growth period of ~7 h for adaptation after inoculation, half of the cultures for each expression plasmid were induced (0.01 mM 3-MB in EtOH) upon reaching an OD_600_ of 0.1. Up to this point, the growth rates of all cultures regardless of the plasmid were the same with an average of 0.545 ± 0.040 h^−1^. Again, the exponential growth continued at the same growth rate for about 3 h. After 10 h upon reaching the late exponential growth phase, the growth rates decreased with uninduced cultures harboring pSEVA438-*teaB* having a growth rate at 0.380 ± 0.013 h^−1^ and the uninduced cultures harboring pSEVA438-*teaC* having a growth rate at 0.381 ± 0.013 h^−1^. Even though the strains have different expression plasmids, the uninduced growth rates were essentially the same. This suggests a very tight control of heterologous expression using the XylS/Pm promoter system in *H. elongata* with a low basal expression similar to *P. putida*, from which this expression system originates (Kessler et al., 1994; Gawin et al., 2017). Surprisingly, the cultures expressing *teaB* also grew at a similar, only slightly reduced rate (0.328 ± 0.021 h^−1^) compared to the uninduced cultures. In contrast, the expression of *teaC* caused a drastic drop to about half of the growth rate (0.189 ± 0.047 h^−1^). The synthesis of TeaC seems to impose a much higher burden on the cell compared to the small subunit TeaB. The concentration of secreted ectoine per dry weight into the medium after 13 h was compared for the *teaB, teaC*, and *teaBC* expression from both batch experiments. Therefore, the ectoine concentration was normed using the uninduced references. As shown in Figure 5B, TeaC facilitates almost 4–5 times the amount that is secreted with either TeaB or TeaBC. But it is not entirely clear how the overexpression of *teaC* impacts cell viability as the growth rate is extremely reduced to about 50%. Additionally, the measured ectoine titers for TeaB and TeaC after 30 h show no significant difference. The final ectoine titer achieved for TeaB was 0.259 ± 0.022 g/L and for TeaC 0.278 ± 0.03 g/L. For the uninduced references lower titers of 0.163 ± 0.003 g/L (TeaB) and 0.163 ± 0.005 g/L (TeaC) were determined. It is possible that the expression of TeaC leads to a loss of membrane integrity. Cell lysis upon induction could explain the strong increase of extracellular ectoine after 13 h. However, as *teaC*-overexpressing cells produce less cell mass due to lysis, *teaB*-overexpressing cells catch up until no differences are found in ectoine titers after 30 h.

## 4. Conclusions

This work has shown two different approaches to increase the synthesis and excretion of ectoine in *H. elongata*. These two strategies clearly complement each other, but were implemented separately to assess their viability. Both approaches have clearly shown to be able to increase the flux toward ectoine and to be promising steps toward strain improvement. Moreover, the phenotypes of the strains created provide further insight on the regulation of intracellular ectoine levels and salt adaptation in *H. elongata*. All the strains were created from a parental strain (KB2.13) that leaks ectoine as a result of a deletion of the TRAP transporter TeaABC.

**Figure 6:**
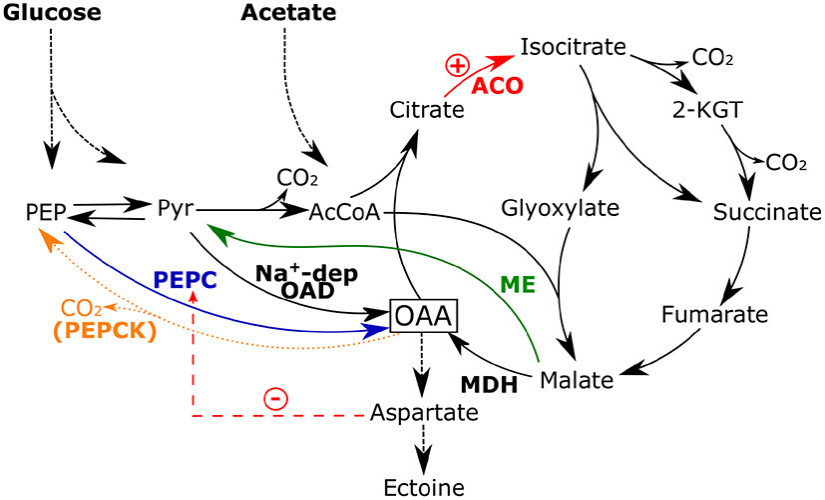
Fluxes around the PEP-PYR-OAA node in KH1.1 caused by the deletion of PEPCK (orange, dashed line). An increase in aspartate could lead to the inhibition of PEPC. RNA-Seq revealed the upregulation of ACO in this mutant, which could be linked to an increased flux through the glyoxylate shunt. The flux toward the TCA cycle might hinder ME activity due to mass action. Thus, MAL could be increasingly channeled toward oxaloacetate *via* MDH.

The first strategy was to increase the supply of oxaloacetate as a central precursor for ectoine synthesis. This was implemented by disrupting the PEP-PYR-OAD node (see Figure 6) that connects glycolysis and the TCA cycle, which has been described as a major switching point for the flux distribution within carbon metabolism (Sauer and Eikmanns, 2005). *H. elongata* utilizes various reactions to adequately split the available carbon flux between feeding the catabolic section of the TCA cycle and the anaplerotic reactions that replenish the carbon skeletons lost to anabolic processes. The deletion of the gene for PEPCK, *pckA*, resulted in a strain (KH1.1) that not only secretes ectoine at a higher rate, but also accumulates it in a higher concentration in the cytoplasm. The subsequent deletion of NADP-dependent ME (MaeB) in KH1.1 resulted in a new strain (KH2.1) which grew at similar rates on glucose and acetate as its parental strain, but had considerably shorter lag phases. Moreover, strain KH3.1 (lacking PckA and Ppc) also had intracellular ectoine concentrations different from the previously mentioned KH1.1 and the parental KB2.13. The fact that intracellular ectoine levels, normally strictly controlled, are significantly different between these strains indicates that the mechanisms controlling ectoine levels in the wild type are no longer functional in all the strains discussed above, probably including the original KB2.13. This has important implications since it simplifies further improvement on ectoine production and supports the hypothesis that the circulation of ectoine between cytoplasm and periplasm fulfils a regulatory function. If PHB accumulation is confirmed in the two strains, a further improvement could be easily obtained from disrupting the PHB synthesis pathway.

The second strategy, increasing ectoine export, also proved able to increase the flux through the pathway and therefore enhance production. This strategy was implemented through overexpression of the TeaBC channel. It is noteworthy that, even though this implementation is not optimal due to the potential coupling between ectoine export and sodium extrusion, it still resulted in a clear improvement of ectoine secretion. This promises high rewards for further work on engineering a more efficient transporter.

## Data availability statement

The RNAseq data generated in this study has been uploaded to SRA and can be accessed under the accession number PRJNA803715.

## Author contributions

AM-S, AK, and HK obtained the funding. HK, KH, and AM-S conceptualized the research. AM-S, KP-G, and KH supervised the work. KH, MO, and NS carried out the experiments. KH and AM-S analyzed the data. KH wrote the first draft of the manuscript. AM-S and HK participated in the elaboration of subsequent versions. All authors read and approved the final version of the manuscript.

## Funding

This work has been funded by the German Federal Ministry of Education and Research (BMBF) through project HOBBIT (031B03).

## Conflict of interest

Authors KH, KP-G, AK, and AM-S are the authors of European patent application EP3833753 (A1)—2021-06-16. An improved method to produce chemical compounds derived from oxaloacetate by microorganisms. The remaining authors declare that the research was conducted in the absence of any commercial or financial relationships that could be construed as a potential conflict of interest.

## Publisher's note

All claims expressed in this article are solely those of the authors and do not necessarily represent those of their affiliated organizations, or those of the publisher, the editors and the reviewers. Any product that may be evaluated in this article, or claim that may be made by its manufacturer, is not guaranteed or endorsed by the publisher.

## Supplementary material

Supplementary Table 1 Summary of all used oligonucleotides in this work.

Supplementary Table 2 TPM data in pseudocounts [log_2_(*n*+1)] as averages of three biological replicates.

Supplementary Table 3 DESeq results comparing KB2.13 vs Wild type *Halomonas elongata*.

Supplementary Table 4 DESeq results comparing KH1.1 vs KB2.13.

Supplementary Table 5 Clusters.
