# Supplementary material for: Metabolic engineering of Halomonas elongata: Ectoine secretion is increased by demand and supply driven approaches
Source: Front Microbiol. 2022 Aug 25;13:968983. doi: 10.3389/fmicb.2022.968983 (PMC9453808; doi:10.3389/fmicb.2022.968983)
Supplement: Supplementary Table 5 — Clusters. [file Data_Sheet_5.PDF]

Table S1: Summary of all used oligonucleotides in this work.

Oligonucleotide	Description	Sequence $5' \rightarrow 3'$
438 teaBC fwdP	Forward primer (lower case, black) for PCR amplification of the *teaB* gene in *H. elongata* for Gibson Assembly cloning into pSEVA438 (GA Overhang in upper case) including a synthetic ribosomal binding site (grey) and regeneration of the restriction site SacI (lower case, underlined)	GCCGCGCGAATTCgagctcaggaggcttcatatgaccgacgaagaagaag
438 teaBC revP	Reverse primer (lower case, black) for PCR amplification of the *teaC* gene in *H. elongata* for Gibson Assembly cloning into pSEVA438 (GA Overhang in upper case) with regeneration of the restriction site HindIII (lower case, underlined)	CAGTCACGACGCGGCCGCaagcttttacctgaaacgcagatcg
438 teaB revP	Reverse primer (lower case, black) for PCR amplification of the *teaB* gene in *H. elongata* for Gibson Assembly cloning into pSEVA438 (GA Overhang in upper case) with regeneration of the restriction site PstI (lower case, underlined)	GCGGCCGCAAGCTTGCATGCctgcagtcagacttccgtttccgtatcc
438 teaC fwdP	Forward primer (lower case, black) for PCR amplification of the *teaC* gene in *H. elongata* for Gibson Assembly cloning into pSEVA438 (GA Overhang in upper case) including a synthetic ribosomal binding site (grey) and regeneration of the restriction site SacI (lower case, underlined)	CCGCGCGAATTCgagctcaggaggcttcatatgacgacaataatggttgc
SW16	Forward primer located in T1 before MCS of pSEVA constructs	gcctttcgttttatttgatgcct
SW17	Reverse primer located in T0 after MCS of pSEVA constructs	ggatctatcaacaggagtccaag
pSW_F	Forward primer for verification of pSW-2 presence	ggacgcttcgctgaaaacta
pSW_R	Reverse primer for verification of pSW-2 presence	aacgtcgtgactgggaaaac
orient_primer	Primer located on the backbone of pSEVA212S and pSEVA511CS to determine the orientation of co-integrates	cgccagggttttcccagtcac
pckA-pSEVA212S-F	Construction of pSEVA212S-Δ*pckA*::Sm^R via Gibson Assembly (Primer sequence in lower case, GA Overhang in upper case) for the marker replacement of *pckA* with a Sm^R cassette	ATCCCCGGGTACCGAGCTCGgatgtctggaagattacttcagc
pckA-up-R		GTCAAGGTTCgtcgattcctgggccttg
pckA-SmR-F		AGGAATCGACgaaccttgaccgaacgcag
pckA-SmR-R		CTCGGCAGGCttatttgccgactaccttggtg
pckA-down-F		CGGCAAATAAgcctgccgagggcgacaac
pckA-pSEVA212S-R		AGGGATAACAGGGTAATCTGcaggtccagatgatcgcgacaagg
pckA-up-Del-R	Construction of Δ*pckA* via Gibson Assembly (Primer sequence in lower case, GA Overhang in upper case) for the deletion of *pckA*, pckA-up-Del-R used together with pckA-pSEVA212S-F and pckA-down-Del-F with pckA-pSEVA212S-R	CTCGGCAGGCgtcgattcctgggccttg
pckA-down-Del-F		AGGAATCGACgcctgccgagggcgacaac
SeqP_DelpckA_fwd	Forward primer upstream of Δ*pckA* and the used upstream flank used for sequencing	gaagggcagatcaccatcac
SeqP_DelpckA_rev	Reverse primer downstream of Δ*pckA* and the used downstream flank used for sequencing gtcgagcagttccacggtag	
Del-maeB up-pSEVA F	Construction of pSEVA212S-Δ*maeB*::Sm^R via Gibson Assembly (Primer sequence in lower case, GA Overhang in upper case) for the marker replacement of *maeB* with a Sm^R cassette	ATCCCCGGGTACCGAGCtcgtccgaagggtacgcgtaccc
Del-maeB up-Sm R		GTCAAGGTTCgctgaggatccgatctgcgattc
Del-maeB up-Sm F		GATCCTCAGCgaaccttgaccgaacgcagc
Del-maeB Sm-down R		TTAAGCAGTAttatttgccgactaccttggtg
Del-maeB Sm-down F		CGGCAAATAAtactgcttaaatgcctgcacgtgc
Del-maeB down-pSEVA R		AGGGATAACAGGGTAATCTGctgcgcggcgagatacgtca
Del-maeB up-down R	Construction of pSEVA212S-Δ*maeB* via Gibson Assembly (Primer sequence in lower case, GA Overhang in upper case) for the deletion of *pckA*, Del-maeB up-down R used together with Del-maeB up-pSEVA F and Del-maeB up-down F with Del-maeB down-pSEVA R	TTAAGCAGTAgctgaggatccgatctgcgattc
Del-maeB up-down F		GATCCTCAGCtactgcttaaatgcctgcacgtg
gHe_M_F	Forward primer upstream of *maeB* and the used upstream flank used for sequencing	ttggacagcgggagttcg
gHe_M_R	Reverse primer downstream of *maeB* and the used downstream flank used for sequencing	aacaccgacagccagacatt
